# Lysosomal chymotrypsin induces mitochondrial fission in apoptotic cells by proteolytic activation of calcineurin

**DOI:** 10.1007/s13238-014-0085-5

**Published:** 2014-07-22

**Authors:** Qianqian Chen, Juan Zhang, Kai Zhao, Wei Li, Qi Miao, Yang Sun, Xingyu Zhao, Taotao Wei, Fuyu Yang

**Affiliations:** 1 National Laboratory of Biomacromolecules, Institute of Biophysics, Chinese Academy of Sciences, Beijing, 100101 China; 2 University of Chinese Academy of Sciences, Beijing, 100049 China; 3 State Key Laboratory of Virology, Wuhan Institute of Virology, Chinese Academy of Sciences, Wuhan, 430071 China

**Dear** **Editor,**

Apoptosis is a fundamental physiological process in mammals in which cells die by activating a suicide mechanism. The mitochondria are one of the major checkpoints in apoptotic regulation because they serve as sensors and amplifiers of cellular damage (Green and Kroemer, 2004). After mitochondrial outer membrane permeabilization (MOMP), the mitochondria release a number of factors that are critically involved in cell death signaling (Tait and Green, 2010). Bcl-2 family members are regarded as the key regulators of mitochondria-dependent apoptosis (Moldoveanu et al., 2014); however, dynamin-related protein 1 (Drp1), which orchestrates mitochondrial fission, also participates in apoptotic regulation by stimulating Bax oligomerization and thereby enhances MOMP (Montessuit et al., 2010); accordingly, the inhibition of Drp1 blocks mitochondrial fission and inhibits apoptosis (Cassidy-Stone et al., 2008).

During mitochondrial fission, Drp1 assembles from the cytosol onto the mitochondria at focal sites of division, forming spiral chains around membrane constriction sites. It has been well documented that the phosphorylation/dephosphorylation of Drp1 may act as a molecular switch to “turn on” or “turn off” mitochondrial fission (Chang and Blackstone, 2010). The phosphorylation of Drp1 at Ser 656 by cyclic-AMP-dependent protein kinase (PKA) induces mitochondrial elongation, whereas the dephosphorylation of Ser 656 by calcineurin promotes mitochondrial fragmentation (Cribbs and Strack, 2007). Calcineurin is a calcium- and calmodulin-dependent phosphatase. In apoptotic cells, the fragmentation of depolarized mitochondria depends on Ca^2+^-evoked, calcineurin-mediated dephosphorylation of Drp1 at its conserved serine 637 site (Cereghetti et al., 2008). The importance of calcineurin in mitochondrial fission is also supported by the findings that an inhibitor of calcineurin (Cereghetti et al., 2010) or the use of a miRNA targeting calcineurin (Wang et al., 2011) regulates mitochondrial fission by modulating Drp1 dephosphorylation and translocation. In this study, we explored an uncanonical, lysosomal chymotrypsin-mediated activation mechanism of calcineurin that leads to Drp1-mediated mitochondrial fission in apoptotic cells.

We have previously reported that chymotrypsin is not only a digestive enzyme secreted by the pancreas but also expressed widely in rat tissues (Zhao et al., 2010) and cached in lysosomes (Miao et al., 2008). However, the expression and subcellular localization of chymotrypsin in cells with human origin remain unclear. Using immunofluorescence, we found that chymotrypsin was colocalized with the lysosomal marker protein LAMP1 in human neuroblastoma SH-SY5Y cells (Fig. 1A). Upon LeuLeuOMe treatment, which induced lysosomal membrane permeabilization (LMP) directly, lysosomal chymotrypsin was relocated to the cytosol. The induction of LMP triggered apoptosis in SH-SY5Y cells, which was evidenced by the release of mitochondrial cytochrome *c* (Fig. 1B), the cleavage of PARP by activated caspase 3 (Fig. 1C), and the increase in the percentage of apoptotic cells with sub-G_1_ DNA content (Fig. 1D). The pretreatment of cells with TPCK, which is a specific chymotrypsin inhibitor, effectively inhibited caspase 3 activation and prevented apoptosis, suggesting that lysosomal chymotrypsin may be responsible for the LMP- triggered apoptosis. To further confirm that the translocation of chymotrypsin to the cytosol was sufficient to induce apoptosis, we introduced recombinant human chymotrypsin into the cytosol of SH-SY5Y cells with the BioPorter reagent and found that the intracellular delivery of chymotrypsin significantly potentiated apoptosis, suggesting that chymotrypsin plays a proapoptotic role (Fig. 1E).

**Figure 1:**
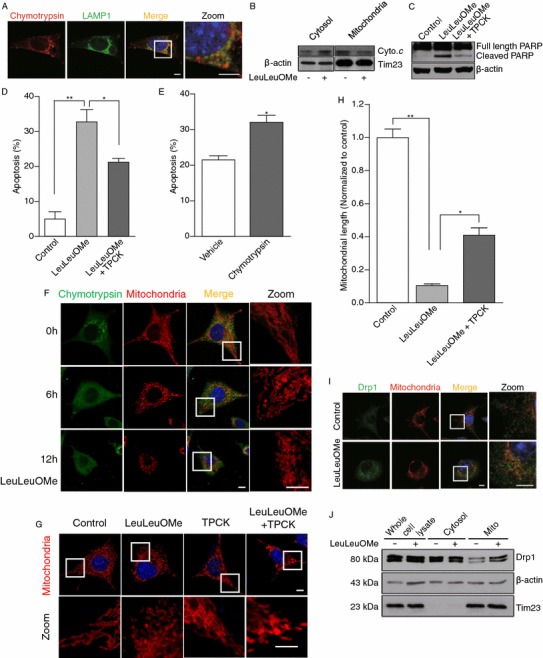
Redistribution of lysosomal chymotrypsin accounts for mitochondrial fission in apoptotic cells. (A) Immunofluorescence visualization of the lysosomal localization of chymotrypsin. Scale bar, 10 μm. (B) Redistribution of mitochondrial cytochrome *c* to the cytosol upon LeuLeuOMe exposure. (C) Activation of caspase 3 upon LeuLeuOMe exposure. (D) Increase in the percentage of apoptotic cells upon LeuLeuOMe exposure. *P < 0.05; **P < 0.01. (E) Intracellular delivery of recombinant chymotrypsin induces apoptosis in SH-SY5Y cells. *P < 0.05. (F) LeuLeuOMe-induced redistribution of lysosomal chymotrypsin and consequent mitochondrial fission in SH-SY5Y cells. (G) Inhibition of chymotrypsin activity blocks mitochondrial fission. (H) Statistical analysis of mitochondrial length in 20 randomly selected cells. *P < 0.05; **P < 0.01. (I) Translocation of Drp1 to the mitochondria, as observed by immunofluorescence. (J) Translocation of Drp1 to the mitochondria, as measured by cell fractionation

We found that mitochondrial fission was an event that occurs downstream of LMP in early stage apoptotic cells. Confocal microscopic images indicated that the mitochondria tended to fuse when the lysosomes are intact, whereas 6-h LeuLeuOMe treatment, which induced LMP, ultimately led to mitochondrial fission (Fig. 1F). Pretreatment with TPCK partially blocked the fission of mitochondria (Fig. 1G and 1H); however, E64d and pepstatin A, two inhibitors of the lysosomal cathepsins, had no apparent effect on the LeuLeuOMe-induced mitochondrial fission and apoptosis (data not shown**)**. These data suggested the involvement of chymotrypsin in mitochondrial fission during apoptosis.

Mitochondrial fission depends on the translocation of cytoplasmic Drp1 to mitochondria, where it binds to Fis1, oligomerizes, and constricts the organelle, ultimately leading to mitochondrial fission. After LeuLeuOMe exposure, which triggered LMP, the confocal microscopic images showed that cytosolic Drp1 was translocated to the mitochondria (Fig. 1I). Drp1 translocation was further confirmed by cell fractionation and immunoblot analysis of the cytosolic and mitochondrial Drp1 levels. As shown in Fig. 1J, the Drp1 level was decreased in the cytosolic fraction and increased in the mitochondrial fraction in cells treated with LeuLeuOMe compared with the control cells.

Because the phosphatase calcineurin regulates the translocation of Drp1, we then asked whether chymotrypsin affects Drp1 translocation and mitochondrial fission via calcineurin-related mechanisms. Calcineurin is a multi-domain phosphatase with an autoinhibitory domain (AID; residues 465–487) and a calmodulin (CaM)-binding domain (CaMBD; residues 392–414) in the C-terminus segment (Fig. 2A) and shows phosphatase activity upon Ca^2+^/CaM binding, which triggers the release of the AID domain from the catalytic active site. However, calcineurin can also be activated or deactivated by proteolysis in apoptotic cells. To test the effects of the relocated lysosomal chymotrypsin on calcineurin, we incubated the SH-SY5Y cell lysate with recombinant chymotrypsin and analyzed the proteolysis of endogenous calcineurin. The immunoblot analysis indicated that chymotrypsin cleaved calcineurin into a ~47-kDa fragment (Fig. 2B, left panel) and a ~13-kDa fragment (Fig. 2B, right panel). Using pNPP as the substrate, we found that the ~47-kDa fragment of cleaved calcineurin showed high phosphatase activity. Notably, the presence of Ca^2+^/CaM showed no additional effect on the activity of truncated calcineurin, suggesting that chymotrypsin cleavage activated the phosphatase activity of calcineurin in a Ca^2+^/CaM-independent manner (Fig. 2C).

**Figure 2:**
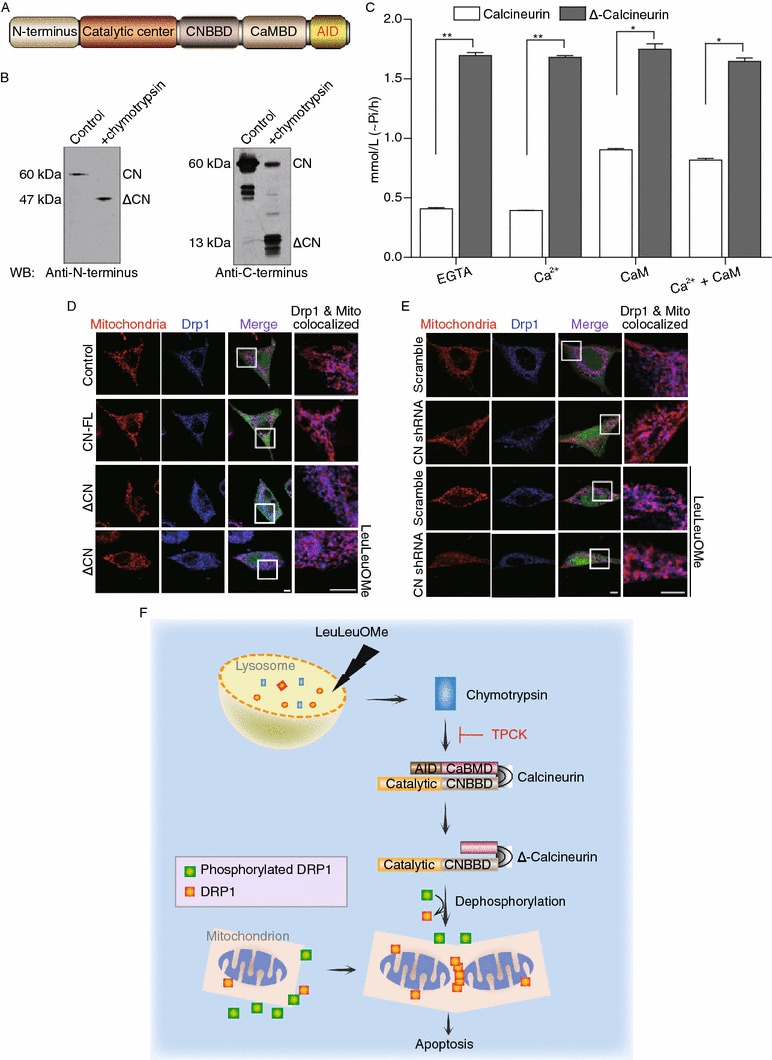
Proteolytic activation of calcineurin mediates mitochondrial fission. (A) Domains of calcineurin. (B) Cleavage of calcineurin by chymotrypsin in SH-SY5Y cells. (C) Proteolytic activation of calcineurin by chymotrypsin. *P < 0.05; **P < 0.01. (D) Truncated calcineurin induces mitochondrial fission and sensitizes cells to apoptotic stimulus. (E) Knockdown of calcineurin by shRNA prevents LeuLeuOMe-induced Drp1 translocation and mitochondrial fission. (F) Schematic diagram of lysosomal chymotrypsin-dependent mitochondrial fission in apoptotic cells

We then attempted to explore the mechanism underlying the proteolytic activation of calcineurin. After chymotrypsin cleavage, a ~47-kDa fragment could be detected with an N-terminus-specific antibody (Chemicon AB1696), and a ~13-kDa fragment could be detected with a C-terminus-specific antibody (BD Pharmingen 65061A). Thus, we reasoned that the cleavage site of calcineurin by chymotrypsin should be located at its regulatory CaMBD, which results in the removal of the AID domain. However, the catalytic domain and the calcineurin B-binding domain (CNBBD) remained intact in the ~47-kDa fragment of truncated calcineurin. As expected, the ~47-kDa fragment of truncated calcineurin showed high phosphatase activity independent of Ca^2+^/CaM and was thus capable of catalyzing the dephosphorylation of various proteins, including Drp1.

To confirm that the proteolytic activation of calcineurin affects the translocation of Drp1 and mitochondrial fission, we transfected SH-SY5Y cells with GFP-tagged full-length (CN-FL) or truncated calcineurin (ΔCN; activated form without AID domain) and observed the morphology of the mitochondria. The results shown in Fig. 2D clearly indicated that full-length calcineurin had no apparent effect on the translocation of Drp1 or on mitochondrial dynamics. In contrast, truncated calcineurin induced the translocation of cytosolic Drp1 to the mitochondria and triggered significant mitochondrial fission. Moreover, when the cells transfected with truncated calcineurin were treated with LeuLeuOMe for 4 h, mitochondrial fission was further enhanced. This result indicated that calcineurin activation by chymotrypsin cleavage can regulate mitochondrial dynamics. The importance of calcineurin in mitochondrial fission was further investigated by RNAi studies. When the expression of calcineurin was down-regulated, LeuLeuOMe-induced LMP failed to induce the translocation of Drp1, and mitochondrial fission was also blocked (Fig. 2E), suggesting that the proteolytic activation of calcineurin by chymotrypsin mediates the mitochondrial dynamics.

In the present investigation, we revealed an uncanonical mechanism of lysosomal chymotrypsin-mediated calcineurin activation that bridges the LMP with the mitochondrial dynamics in apoptotic cells. Lysosomes, which are acidic organelles containing dozens of hydrolytic enzymes (Boya, 2012), are tightly linked with apoptosis (Aits and Jaattela, 2013). Signaling pathways that lead to lysosome-dependent apoptosis have been extensively investigated. In certain cell types, cathepsins and/or chymotrypsin rapidly process and thereby activate Bid in the cytosol, which in turn triggers MOMP and results in mitochondria-dependent apoptosis (Zhao et al., 2010; Zhao et al., 2012). However, the involvement of Bid in lysosome- and mitochondria-dependent apoptosis depends on the nature of the apoptotic stimuli and on the cell type. In addition to Bid, calcineurin also connects lysosomal chymotrypsin to mitochondrial destruction. The canonical activation of calcineurin depends on the calcium signal. In apoptotic cells, the Ca^2+^-evoked activation of calcineurin mediates the dephosphorylation of Drp1 at its conserved serine 637 site, triggers the translocation of Drp1, and results in the fragmentation of depolarized mitochondria (Cereghetti et al., 2008). However, the present investigation revealed a novel pathway leading to calcineurin activation and mitochondrial fission. As a result of lysosomal membrane permeabilization, lysosomal chymotrypsin was relocated to the cytosol, where it processed calcineurin at its regulatory Ca^2+^/CaM-binding domain, resulting in the formation of the ~47-kDa fragment with high phosphatase activity independent of Ca^2+^/CaM. The proteolytic activation of calcineurin triggered the translocation of cytosolic Drp1 to the mitochondria, consequently inducing Drp1-mediated mitochondrial fission and culminating in sufficient apoptotic signaling to cause mitochondria-dependent apoptosis (Fig. 2F).

## Electronic supplementary material

Below is the link to the electronic supplementary material.

Supplementary material 1 (PDF 137 kb)
