# Supplementary material for: Lysosomal chymotrypsin induces mitochondrial fission in apoptotic cells by proteolytic activation of calcineurin
Source: Protein Cell. 2014 Jul 22;5(8):643–7. doi: 10.1007/s13238-014-0085-5 (PMC4130920; doi:10.1007/s13238-014-0085-5)
Supplement: Supplementary file 1 — Supplementary material 1 (PDF 137 kb) [file 13238_2014_85_MOESM1_ESM.pdf]

Supplementary materials

Lysosomal Chymotrypsin Induces Mitochondrial Fission in Apoptotic Cells by Proteolytic Activation of Calcineurin

Qianqian Chen[1, 2, #], Juan Zhang[1, #], Kai Zhao[1, #], Wei Li[3], Qi Miao[1], Yang Sun[1], Xingyu Zhao[1], Taotao Wei[1, *] and Fuyu Yang[1, *]

[1] *National Laboratory of Biomacromolecules, Institute of Biophysics, Chinese Academy of Sciences, 15 Datun Road, Chaoyang District, Beijing 100101, China*

[2] *University of Chinese Academy of Sciences, 19 Yuquan Road, Shijingshan District, Beijing 100049, China*

[3] *State Key Laboratory of Virology, Wuhan Institute of Virology, Chinese Academy of Sciences, 44 Xiaohongshan, Wuchang District, Wuhan 430071, China*

[#] QC, JZ and KZ contributed equally to the present investigation.

[*] Correspondence: yangfy@sun5.ibp.ac.cn; weitt@moon.ibp.ac.cn.

Materials and Methods

Cell culture, DNA constructs, and transfection

SH-SY5Y human neuroblastoma cells obtained from ATCC (American Type Culture Collection, Manassas, VA, USA) were cultured in DMEM/F12 (Life Technologies, Grand Island, NY, USA) supplemented with 10% heat-inactivated fetal calf serum, 100 U/ml penicillin, and 100 μg/ml streptomycin at 37°C with 5% CO_2. To construct expression vectors containing full-length or truncated calcineurin, the cDNA for calcineurin was cloned by RT-PCR using human mRNA as the template. The cDNAs for full-length calcineurin and truncated calcineurin (1-410 lacking the C-terminal autoinhibitory domain) were subcloned into the mammalian expression vector pEGFP-C2 (Clontech, Mountain View, CA, USA). The shRNA targeting the calcineurin was provided by RiboBio (Guangzhou, China).

In some experiments SH-SY5Y cells were transfected using Fugene HD reagent (Promega, Madison, WI, USA) according to the manufacturer's instructions.

Expression of recombinant proteins

Recombinant human chymotrypsin was obtained as previously described (Zhao et al., 2010). Recombinant human calcineurin α was expressed and purified as previously described (Mondragon et al., 1997).

Intracellular delivery of recombinant chymotrypsin

Recombinant chymotrypsin was delivered to SH-SY5Y cells using the BioPORTER protein transfection reagent (Gene Therapy Systems; San Diego, CA, USA) as described previously (Zhao et al., 2010).

Immunofluorescence and confocal microscopy

SH-SY5Y neuroblastoma cells on glass coverslips were fixed with 4% paraformaldehyde in PBS, permeabilized with 0.2% Triton X-100, and blocked for 1

h at room temperature with PBS containing 5% goat serum albumin. Cells were incubated with antibodies (anti-chymotrypsin, Santa Cruz Biotechnology, Santa Cruz, CA, USA; anti -Drp-1, Cell Signaling Technology, Danvers, MA, USA) and then incubated with the appropriate secondary antibodies. To visualize mitochondria in living cells, cells were stained with MitoTracker Red CMXRos (Life Technologies) at 37°C for 30 min and washed twice with PBS. Nuclei were stained with DAPI (Sigma-Aldrich, St. Louis, MO, USA). Cells were visualized by Z-Stack imaging with a confocal microscope (Olympus FV1000; Tokyo, Japan) and processed using Fluoview software (Olympus). The length of mitochondria was measured using Image-Pro Plus software (Media Cybernetics, Rockville, MD, USA) as described previously (Zhao et al., 2013).

Immunoblotting

SH-SY5Y cells were lysed in ice-cold RIPA buffer (50 mM Tris-HCl, pH 7.4; 1% NP-40; 150 mM NaCl; 0.25% sodium deoxycholate; 1 mM EDTA; and a protease inhibitor mixture). After ultracentrifugation at 10,000×g for 30 min at 4°C, supernatants containing solubilized proteins were analyzed by immunoblotting. In some experiments, mitochondrial or cytosolic fractions were fractionized by protocols described previously (Zhao et al., 2010). Samples were resolved by SDS-PAGE, transferred to a polyvinylidene fluoride (PVDF) membrane, and blotted with proper primary antibodies (anti-β-actin, Santa Cruz; anti-PARP, anti-cytochrome *c* and anti-Drp1, Cell Signaling Technology; anti- N-terminus of calcineurin, Chemicon, Cambridge, UK; anti-C-terminus of calcineurin, anti-Tim23, BD Bioscience, Sparks, MD, USA. They were then incubated with appropriate peroxidase-conjugated secondary antibodies (Santa Cruz, Santa Cruz, CA, USA) and visualized using a chemiluminescent substrate (GE Amersham Pharmacia ECL; Beijing, China) with

Kodak X-OMAT film (Rochester, NY, USA).

Apoptosis assays

Cells were harvested by mild trypsinization, washed with cold PBS, and fixed with 70% ethanol. Cells were stained with propidium iodide (PI, Sigma-Aldrich, St. Louis, MO, USA), and the percentage of hypodiploid (apoptotic) cells was measured with a FACSCalibur flow cytometer.

In vitro cleavage of endogenous calcineurin

SH-SY5Y cells were lysed in ice-cold buffer (50 mM Tris-HCl, pH 7.4; 150 mM NaCl) by sonication. After ultracentrifugation at 10,000×g for 10 min at 4°C, supernatants containing solubilized proteins were incubated with chymotrypsin at 37 °C for 1 h. Samples were resolved by SDS-PAGE and the specific cleavage of endogenous calcineurin was detected by immunoblotting with antibodies against N- or C-terminus of calcineurin, respectively.

Phosphatase activity assay of calcineurin

Calcineurin activity was assayed by measuring the hydrolysis of *p*-nitrophenyl phosphate (pNPP; Sigma-Aldrich) at 405 nm, as described below. Briefly, the assay buffer consisted of 50 mM Tris–HCl (pH 7.5), 2.5 mM DTT, 20 mM pNPP and one of the following: (i) 1 mM EDTA for measurement of background activity; (ii) 100 nM EGTA, 1 mM Ca^{2+}, 1 mM Mn^{2+} for basal activity; (iii) 100 nM EGTA, 1 mM Mn^{2+}, 0.8 mM calmodulin for calmodulin-stimulated activity or (iv) 100 nM EGTA, 1 mM Ca^{2+}, 1 mM Mn^{2+}, 0.8 mM calmodulin for Ca^{2+}/calmodulin-stimulated activity. Because calcineurin activity is enhanced in the presence of Mn^{2+}, Mn^{2+} was included in the assay buffer for conditions ii, iii and iv to aid detection.

References

Mondragon, A., Griffith, E.C., Sun, L., Xiong, F., Armstrong, C., and Liu, J.O. (1997). Overexpression and purification of human calcineurin alpha from Escherichia

coli and assessment of catalytic functions of residues surrounding the binuclear metal center. Biochemistry 36, 4934-4942.

Zhao, J., Zhang, J., Yu, M., Xie, Y., Huang, Y., Wolff, D.W., Abel, P.W., and Tu, Y. (2013). Mitochondrial dynamics regulates migration and invasion of breast cancer cells. Oncogene 32, 4814-4824.

Zhao, K., Zhao, X., Tu, Y., Miao, Q., Cao, D., Duan, W., Sun, Y., Wang, J., Wei, T., and Yang, F. (2010). Lysosomal chymotrypsin B potentiates apoptosis via cleavage of Bid. Cell Mol Life Sci 67, 2665-2678.
